# Supplementary material for: Dynamic Properties of Di(cyclopentadienecarboxylic Acid) Dimethyl Esters
Source: Int J Mol Sci. 2023 Oct 7;24(19):14980. doi: 10.3390/ijms241914980 (PMC10573197; doi:10.3390/ijms241914980)
Supplement: Supplementary file 1 [file ijms-24-14980-s001.zip › IJMS-2607118-supplementar.pdf]

Dynamic Properties of Di(cyclopentadienecarboxylic Acid) Dimethyl Esters

Alojz Anžlovar *, Damjan Jan Pavlica, David Pahovnik, Ema Žagar

Department of Polymer Chemistry and Technology, Nationall Institute of Chemistry, Hajdrihova 19, SI-1000 Ljubljana, Slovenia
* Correspondence: e-mail: alojz.anzlovar@ki.si; Tel.: +386-1-4760-204

Supplementary Materials

The procedure for the GC-MS study of DCPDME – isomer 3 decomposition into cyclopentadienecarboxylic acid methyl ester

The SPME fiber holder for manual use and 75 µm carboxen-polydimethylsiloxane (CAR/PDMSE) fiber from Supelco (Bellefonte, PA, USA) were used for extraction. The fiber was conditioned at 200 °C for 20 minutes. Twenty-milliliter glass vials containing an aluminium-coated silicone rubber septum (Agilent, Santa Clara, CA, USA) were used. A Kambič SP-120 oven (Semič, Slovenia) was used at 110 °C for sample extraction.

Approximately 1 mg of the solid sample of isomer 3 was sealed in a 20-mL vial. The septum of the vial was pierced with the needle of the SPME device and the fiber was exposed about 1 cm above the sample. The vial with the SPME injector was then placed in the oven at 110 °C for 10 minutes. The fiber was then retracted into the protective sleeve and removed from the HS vial. It was transferred to the injection port of the GC/MS without delay. The fiber was thermally desorbed in the injection port at 160 °C for 10 s, and the GC/MS run was started. The HP-5MS fused silica GC capillary column, 30 m long, 0.25 mm I.D., with 0.25 µm film thickness (Agilent, Santa Clara, CA, USA) was used for chromatographic separation. Helium was used as the carrier gas with a constant flow of 1.0 mL/min. The gas chromatographic conditions were as follows: programmed temperature of the capillary column from 60 °C (2 min hold) at 10 °C/min to 100 °C and at 20 °C/min to 180 °C; split/splitless injector temperature was 160 °C; split ratio was 50:1 and transfer line temperature was 200 °C. The temperature of the EI ion source was maintained at 230 °C. Ionization was performed with a kinetic energy of the impacting electrons of 70 eV. The quadrupole temperature was 150 °C. Mass spectra and reconstructed chromatograms (TIC) were obtained by automated scanning in the mass range m/z from 12 to 350 amu. GC/MS data were processed using *ChemStation* software (Agilent, Santa Clara, CA, USA).

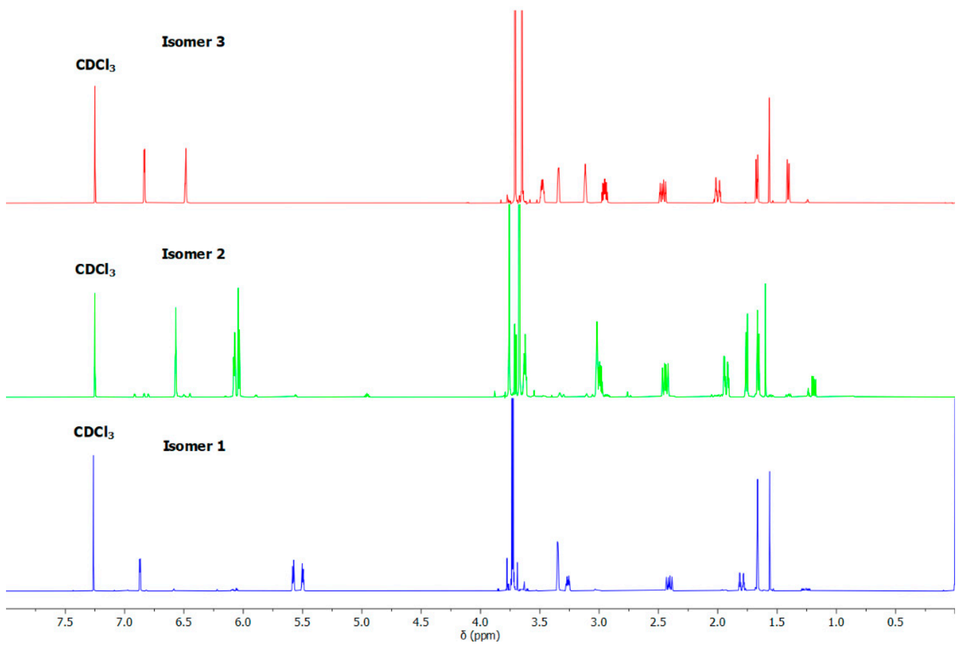

Figure S1. ^{1}H NMR spectra of all three DCPDME isomers in $CDCl_3$.

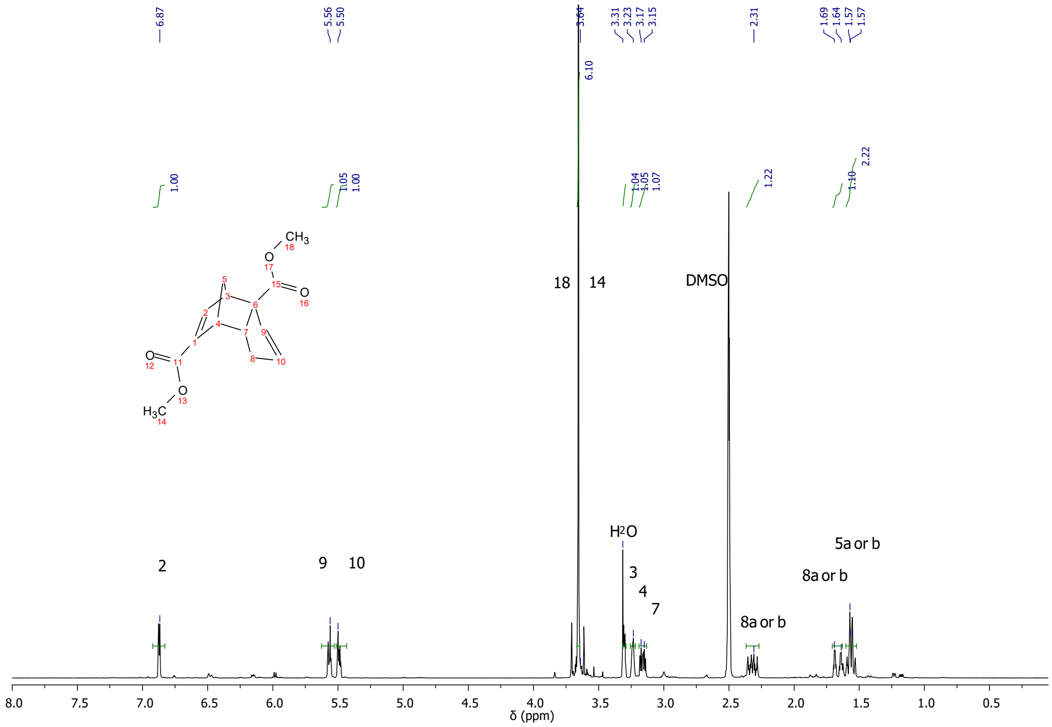

Figure S2. ^{1}H NMR spectrum of DCPDME isomer 1 in DMSO-d_6.

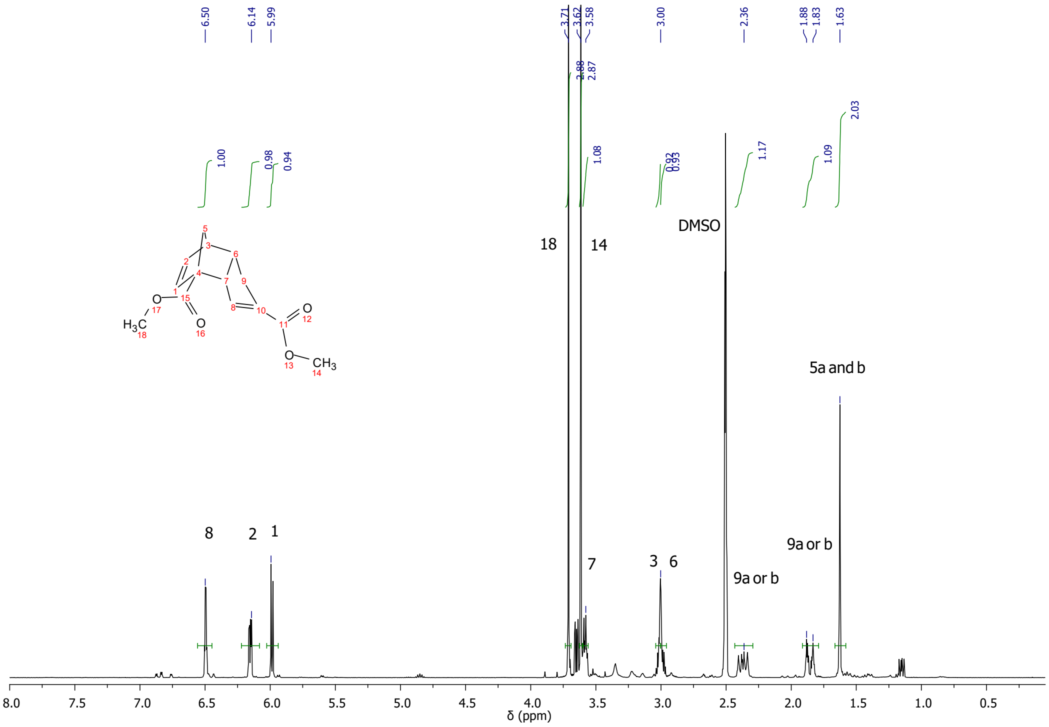

Figure S3. ^{1}H NMR spectrum of DCPDME isomer 2 in DMSO-d_6.

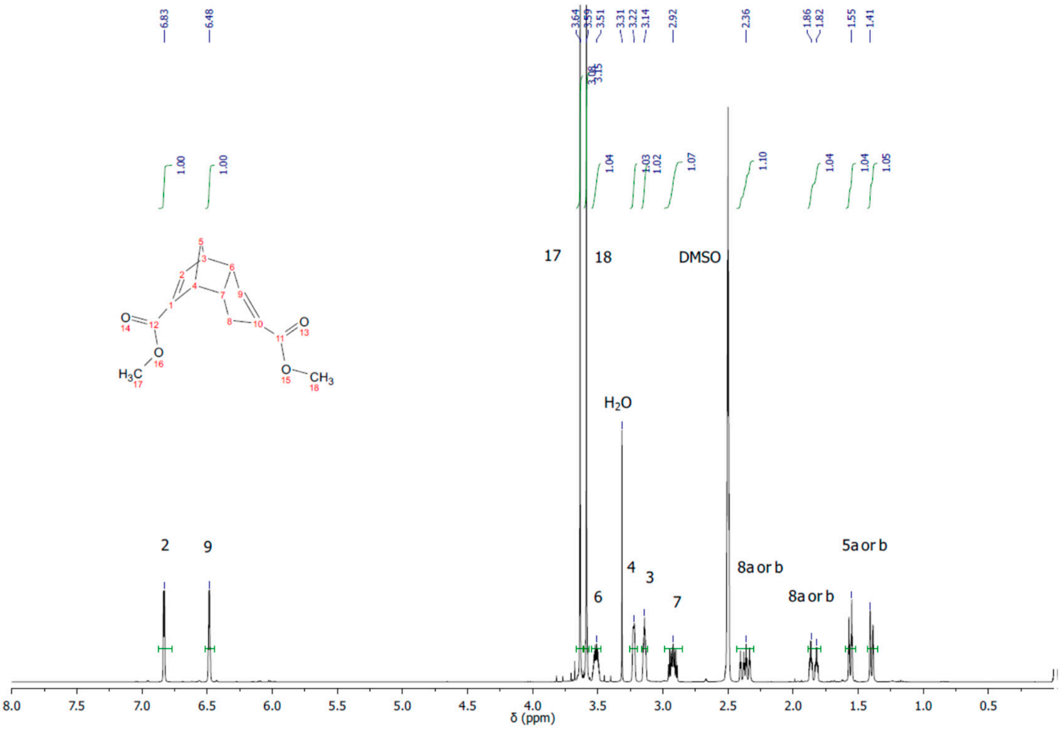

Figure S4. ^{1}H NMR spectrum of DCPDME isomer 3 in DMSO-d_6.

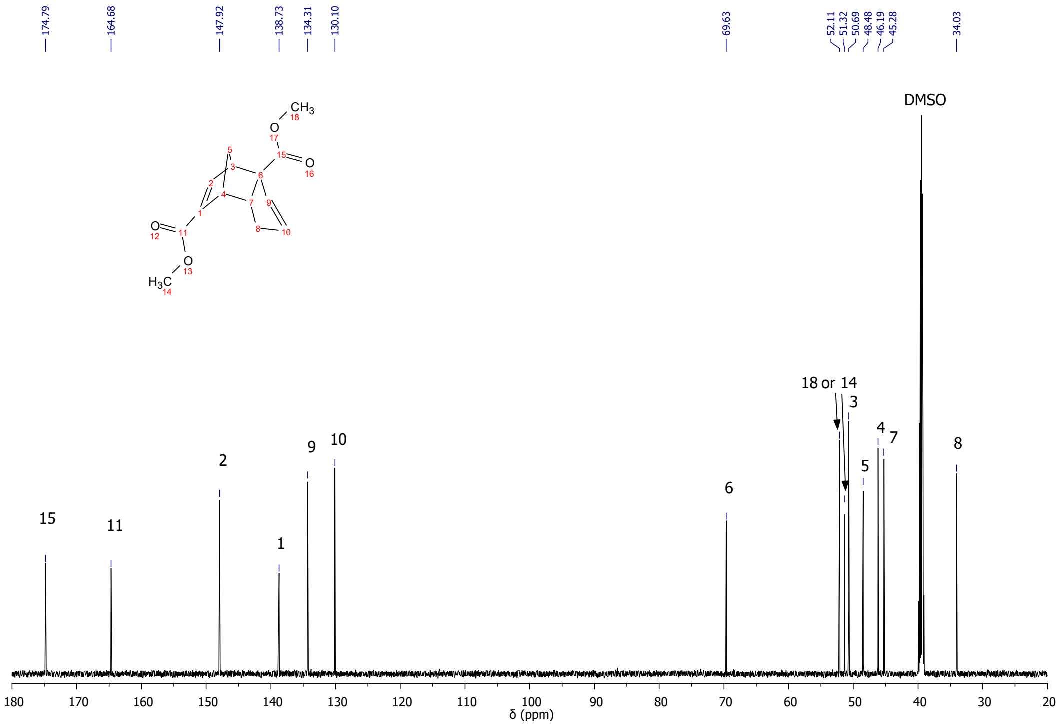

Figure S5. ^{13}C NMR spectrum of DCPDME isomer 1 in DMSO-d_6.

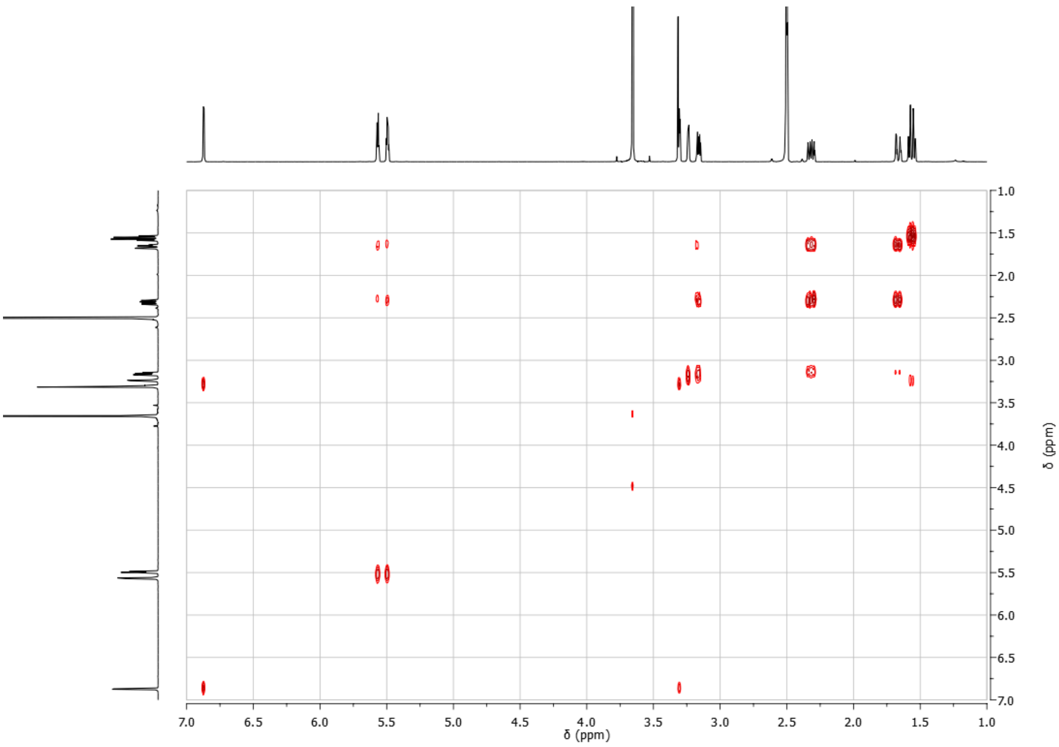

Figure S6. COSY NMR spectrum of DCPDME isomer 1 in DMSO-d_6.

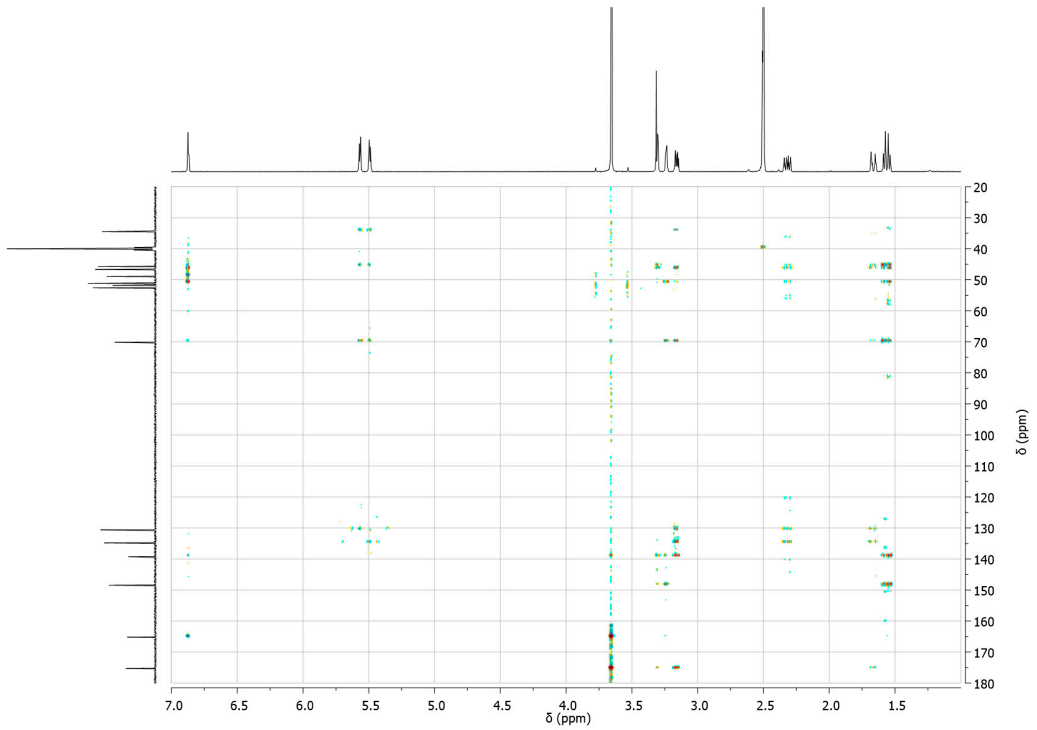

Figure S7. HMBC NMR spectrum of DCPDME isomer 1 in DMSO-d_6.

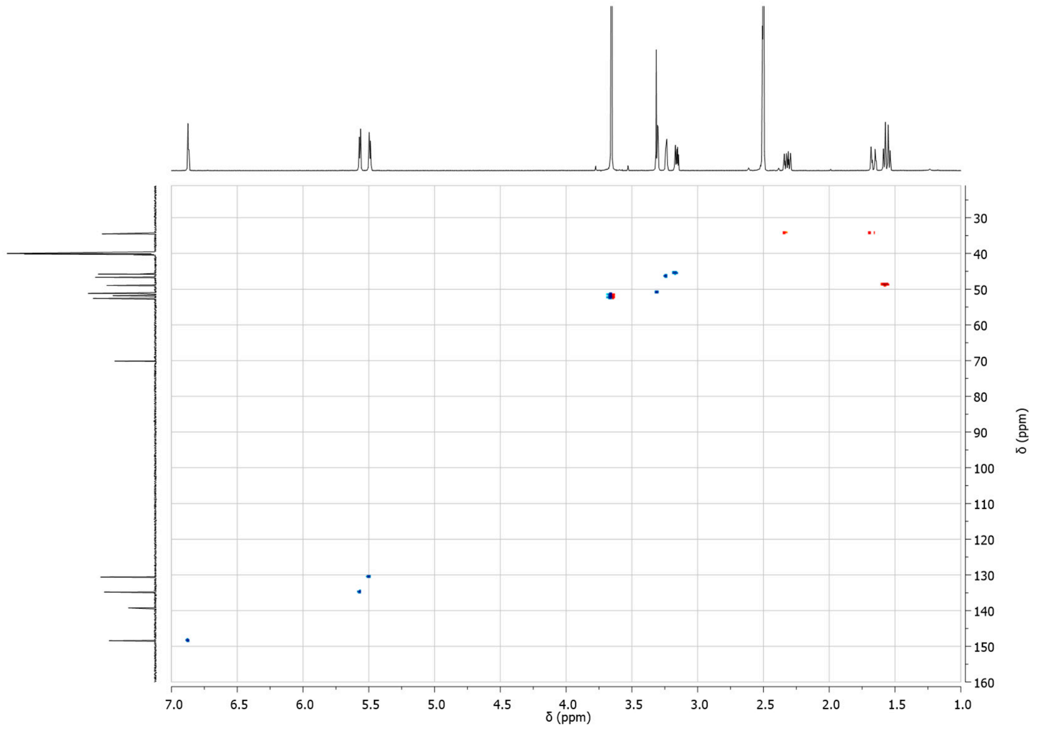

Figure S8. HSQC NMR spectrum of DCPDME isomer 1 in DMSO-d_6.

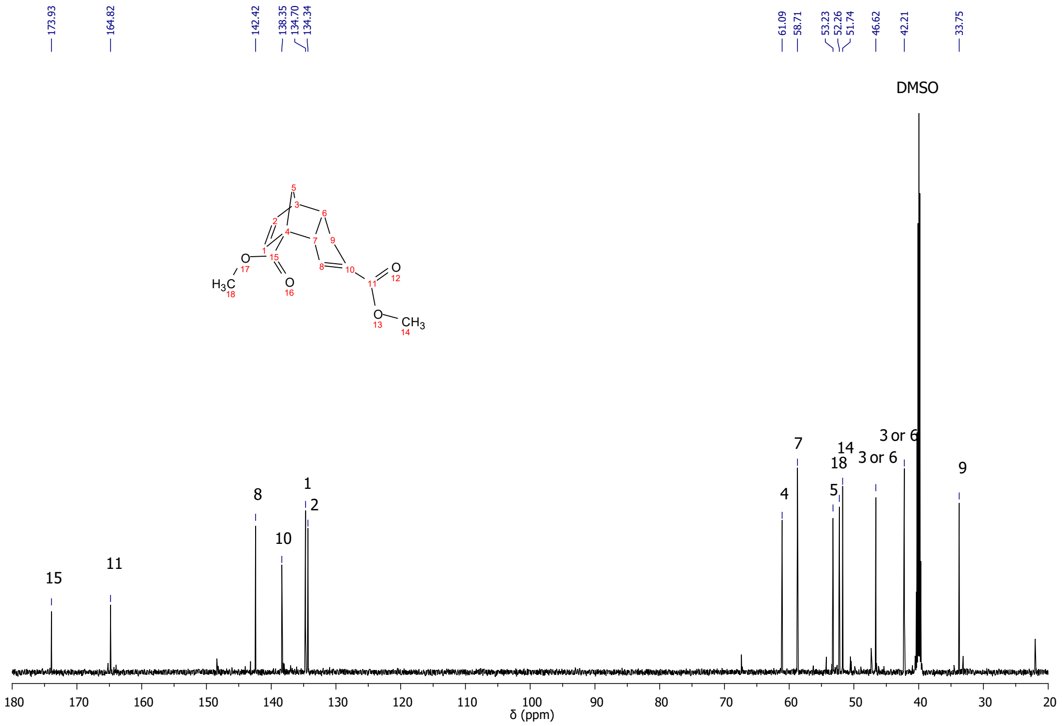

Figure S9. ^{13}C NMR spectrum of DCPDME isomer 2 in DMSO-d_6.

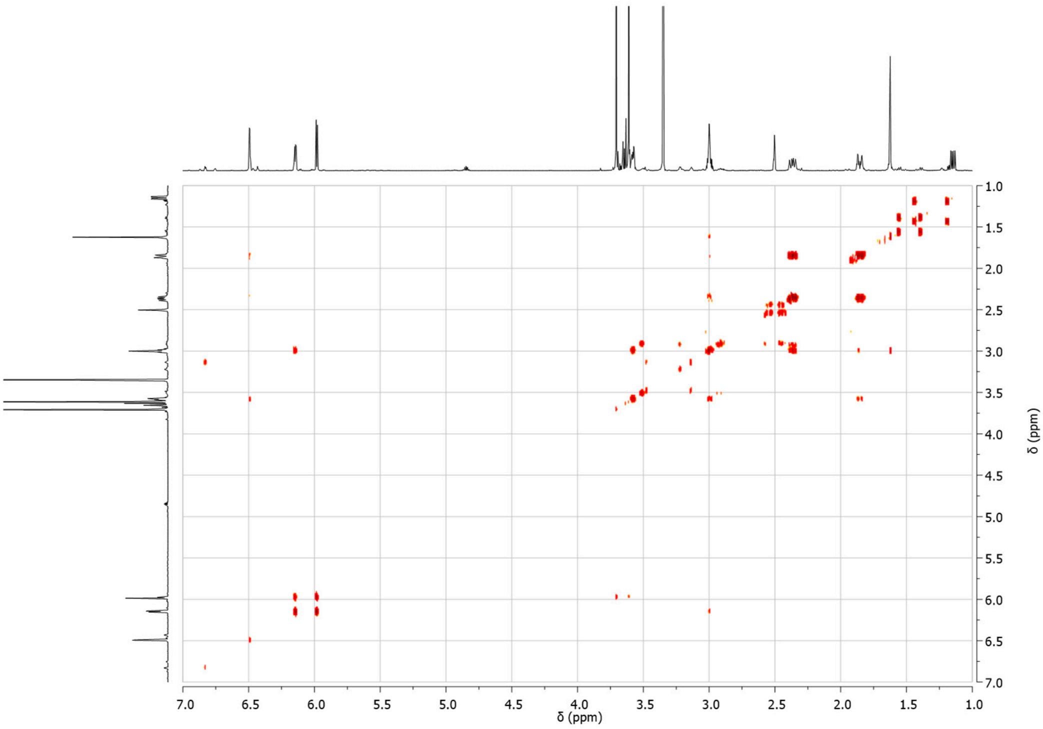

Figure S10. COSY NMR spectrum of DCPDME isomer 2 in DMSO-d_6.

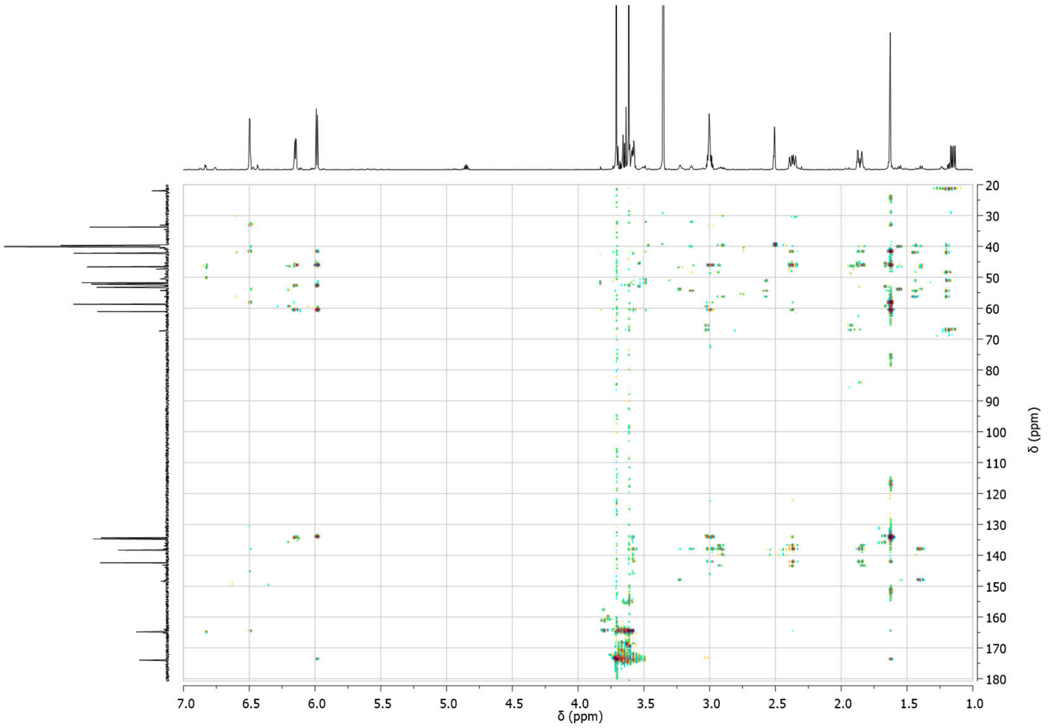

Figure S11. HMBC NMR spectrum of DCPDME isomer 2 in DMSO-d_6.

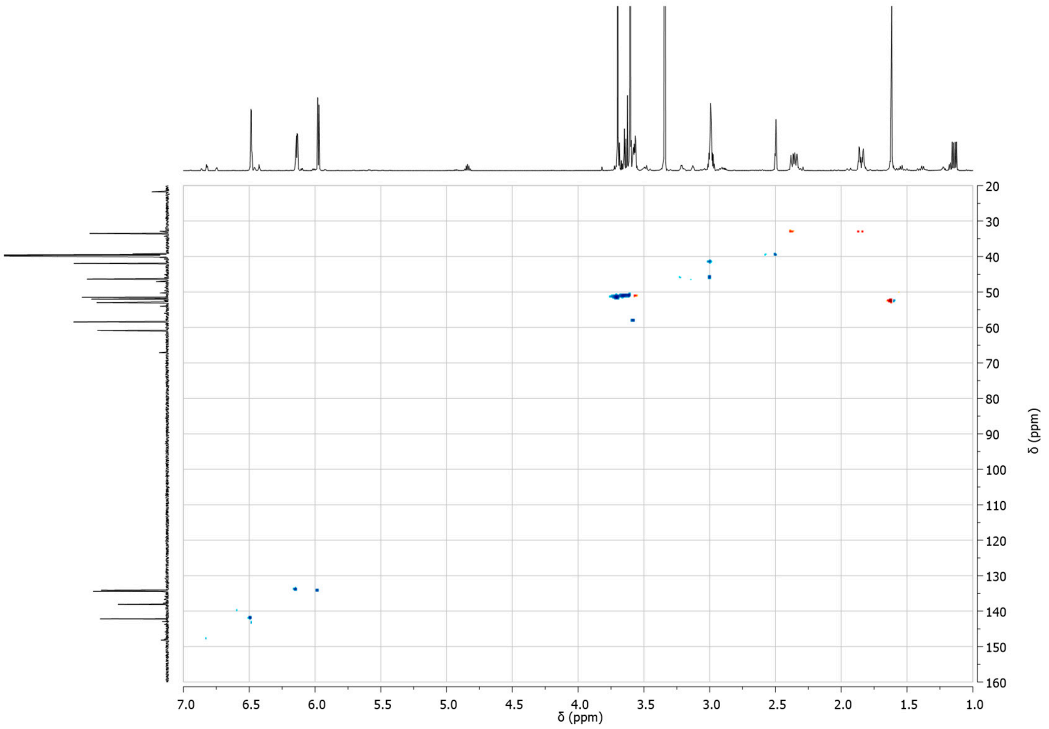

Figure S12. HSQC NMR spectrum of DCPDME isomer 2 in DMSO-d_6.

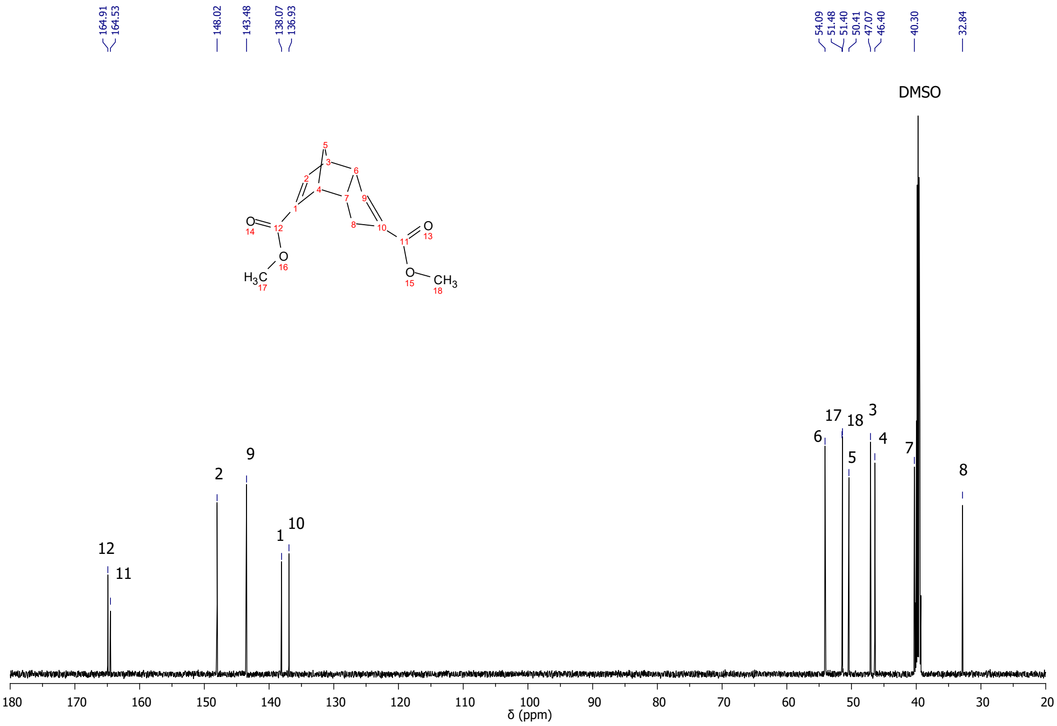

Figure S13. ${}^{13}C$ NMR spectrum of DCPDME isomer 3 in DMSO-d_6.

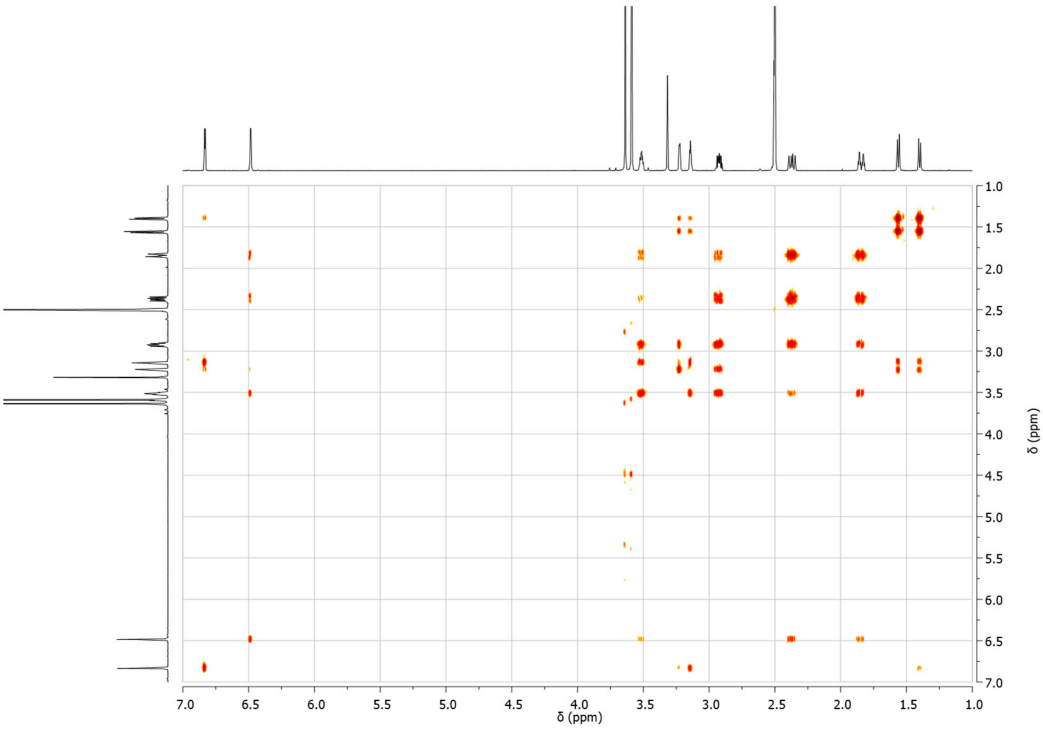

Figure S14. COSY NMR spectrum of DCPDME isomer 3 in DMSO-d_6.

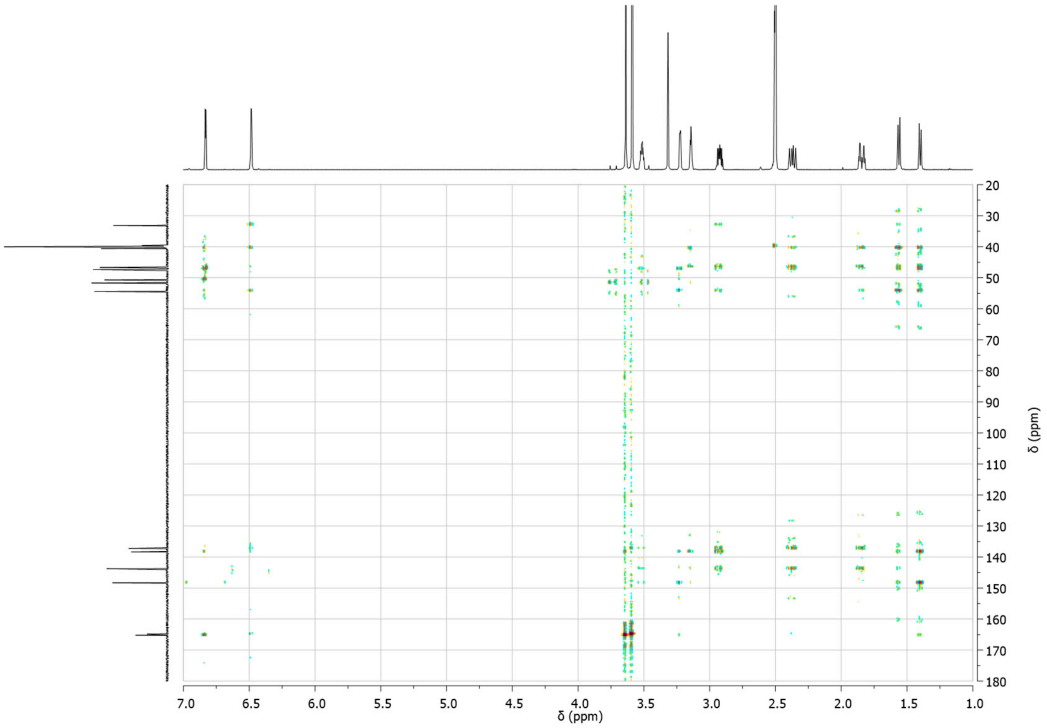

Figure S15. HMBC NMR spectrum of DCPDME isomer 3 in DMSO-d_6.

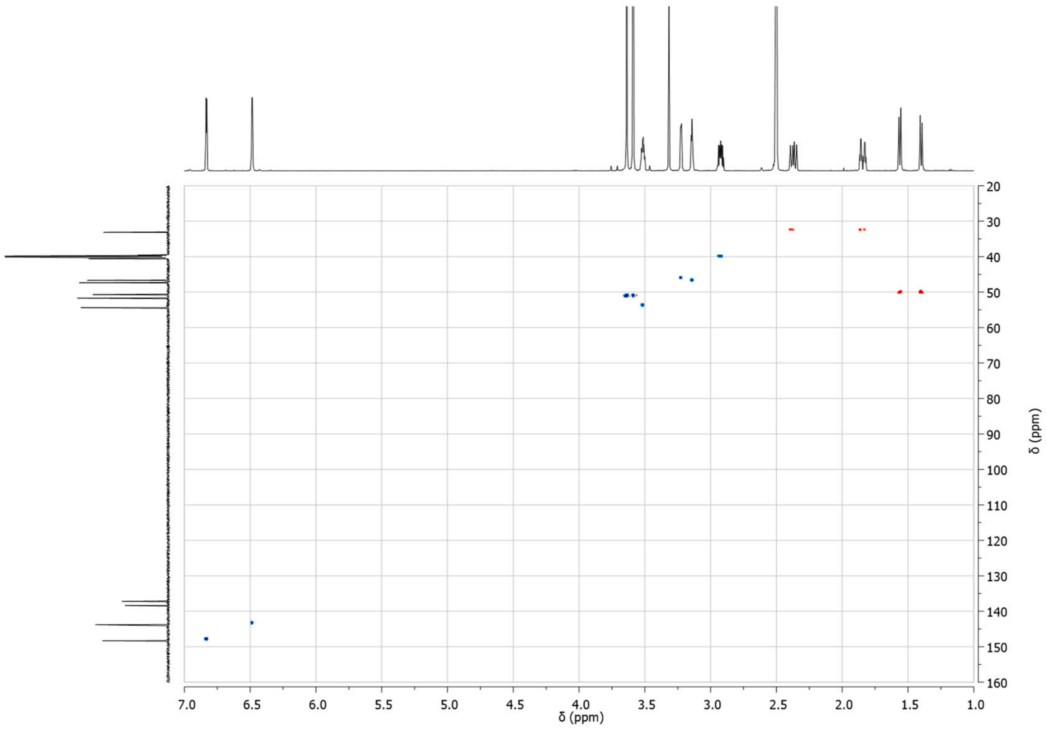

Figure S16. HSQC NMR spectrum of DCPDME isomer 3 in DMSO-d_6.

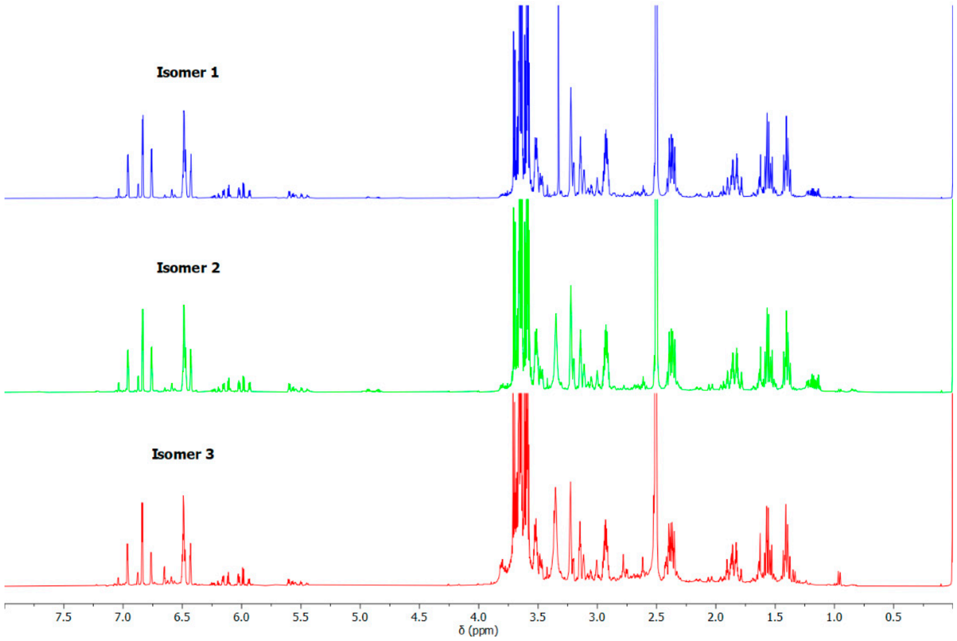

Figure S17. ^{1}H NMR spectra of all three DCPDME isomers after heating to 180 °C for 2 minutes in N_2 in DSC calorimeter.

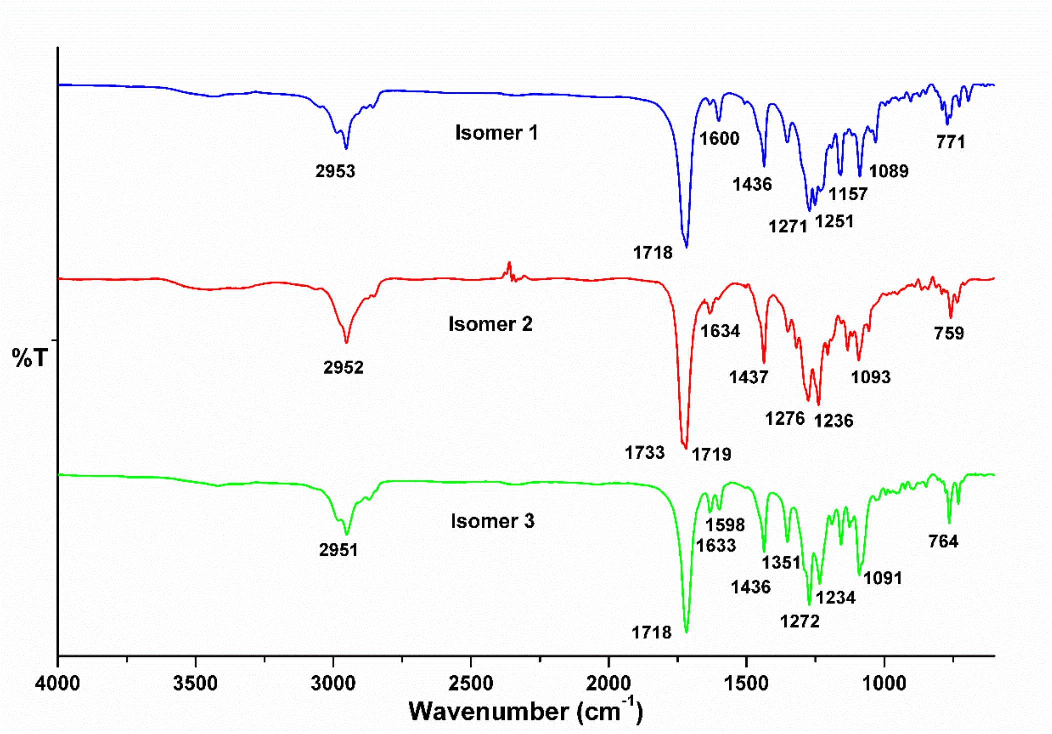

Figure S18. FTIR spectra of all three DCPDME isomers at 125 °C.

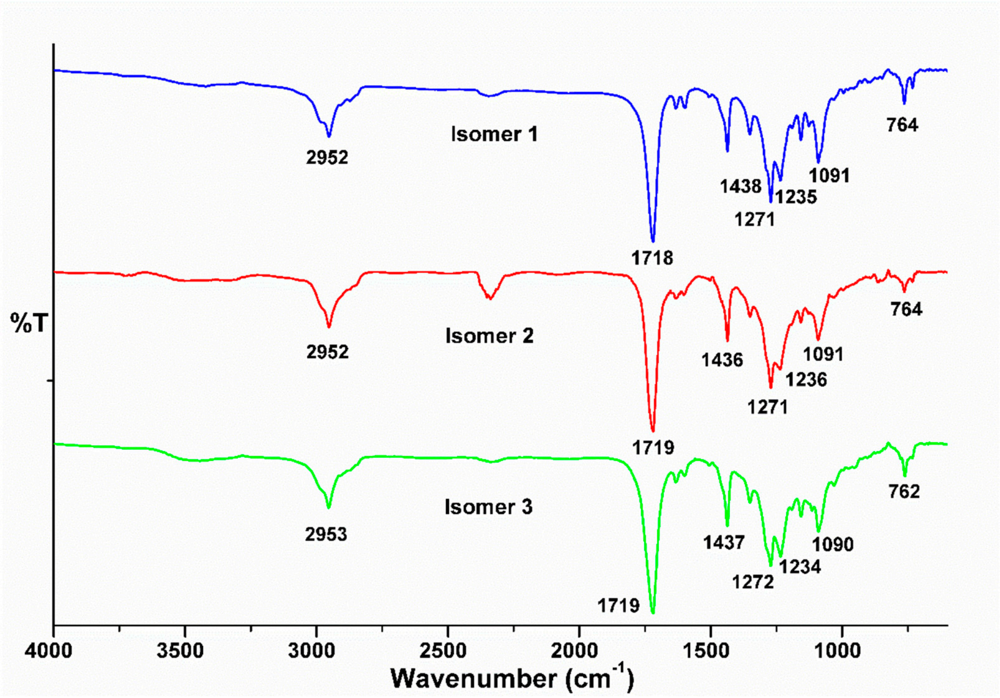

Figure S19. FTIR spectra of all three DCPDME isomers at 140 °C.
